# Men and Women Exhibit a Differential Bias for Processing Movement versus Objects

**DOI:** 10.1371/journal.pone.0032238

**Published:** 2012-03-14

**Authors:** Robert F. McGivern, Brian Adams, Robert J. Handa, Jaime A. Pineda

**Affiliations:** 1 Department of Psychology, San Diego State University, San Diego, California, United States of America; 2 Department of Basic Medical Sciences, University of Arizona College of Medicine, Phoenix, Arizona, United States of America; 3 Department of Cognitive Science and Neuroscience, University of California San Diego, La Jolla, California, United States of America; University of Texas at San Antonio, United States of America

## Abstract

Sex differences in many spatial and verbal tasks appear to reflect an inherent low-level processing bias for movement in males and objects in females. We explored this potential movement/object bias in men and women using a computer task that measured targeting performance and/or color recognition. The targeting task showed a ball moving vertically towards a horizontal line. Before reaching the line, the ball disappeared behind a masking screen, requiring the participant to imagine the movement vector and identify the intersection point. For the color recognition task, the ball briefly changed color before disappearing beneath the mask and participants were required only to identify the color shade. Results showed that targeting accuracy for slow and fast moving balls was significantly better in males compared to females. No sex difference was observed for color shade recognition. We also studied a third, dual attention task comprised of the first two, where the moving ball briefly changed color randomly just before passing beneath the masking screen. When the ball changed color, participants were required only to identify the color shade. If the ball didn't change color, participants estimated the intersection point. Participants in this dual attention condition were first tested with the targeting and color tasks alone and showed results that were similar to the previous groups tested on a single task. However, under the dual attention condition, male accuracy in targeting, as well as color shade recognition, declined significantly compared to their performance when the tasks were tested alone. No significant changes were found in female performance. Finally, reaction times for targeting and color choices in both sexes correlated highly with ball speed, but not accuracy. Overall, these results provide evidence of a sex-related bias in processing objects versus movement, which may reflect sex differences in bottom up versus top-down analytical strategies.

## Introduction

The majority of human cognitive sex differences are broadly categorized as ‘spatial’ or ‘verbal’ [1]. To what degree these differences arise from lower level perceptual processing is unknown, but functional studies of neurophysiological and behavioral sex differences in humans, as well as similarities in cognitive sex differences observed in humans and animals, suggest this possibility [2]. Below we review evidence that the kinds of spatial and verbal skills that show cognitive sex differences may involve differential processing of information involving the dorsal and ventral processing streams that are common to all mammals.

Dorsal and ventral stream cortical processing provide a functional analysis of movement and object recognition, respectively. The dorsal stream provides the basis for conscious and unconscious knowledge of ‘where’ something is in visual space, as well as the tracking of object movement. Ventral stream processing provides information for conscious recognition of ‘what’ something is, including its associated characteristics [3]–[4]. Both cortical streams operate in parallel, with some integration of movement and objects occurring in subdivisions of the dorsal stream that lies anatomically between the two classical pathways [5].

Spatial tasks showing reliable sex differences in men include targeting, maze learning, and dis-embedding tasks such as Rod and Frame and Embedded Figures [6]–[7], all of which involve processing of actual or abstract movement. Some corollaries to these sex differences are observed in animals. For instance, maze learning across species consistently favors males in environments that depend upon employing a cardinal orientation strategy [8]–[10]. Similarly, targeting tasks that involve accuracy in throwing an object also favor males across species [11]–[14]. More complex tasks involving space relations, such as mental rotations, have no direct corollary in animals, but are proposed to partially rely on neural substrates associated with targeting skills [12].

In spite of the categorical label, verbal tasks that show sex differences are not easily related to differences in inherent linguistic ability since men and women exhibit similar writing skills, vocabulary, general fluency, and language and reading comprehension [13]–[14]. Instead, tasks in this category showing sex differences rely on verbal or written expression of knowledge related to objects (or events) and their associated characteristics. These include fluency in naming words beginning with a given letter, autobiographical and episodic memory, and communication skills, all of which favor women [6] [14]. The pattern suggests that females have a broader network of associations among objects than males, which allows for greater verbal elaboration and description. This is consistent with the greater bilateral cortical activation in women, as well greater activation of the left temporal pole, during passive listening to narratives or verbal descriptions related to episodic memory [15]–[16].

More direct evidence of a female advantage related to object processing comes from studies where participants are exposed to incidental visual stimuli and subsequently tested for their recall. Under both real-life and experimental conditions, females exhibit better implicit memory for object recall than males, in addition to showing greater recall of the object location [17]–[19] The unconscious aspect of the female advantage in object recall is emphasized by findings showing that no gender difference is observed when participants know the nature of the task [20]. Interestingly, although object location memory and object identity memory are distinctly different tasks, Voyer et al. observed a .37 Pearson correlation between the two tasks in a sample of 223 participants, suggesting that performance in both may rely on a common mediating process [17]. Choi and L'Hirondelle [21] have proposed that verbal memory may account for the female advantage, which is supported by a number of these kinds of implicit memory studies where no sex difference was observed when the objects were uncommon or not nameable [22]–[24].

The object location memory task favoring females involves a spatial skill [18] [25]–[26] that is distinct from space relation skills inherent to the tasks favoring males such as mental rotation or embedded figures. In the object location task, females are thought to place more reliance on semantic encoding of the object as the primary organizational strategy, with space relations taking a secondary role, whereas males may use space relations as the primary strategy [17] [27]. This kind of sex related pattern can also be observed in studies of navigational strategy. Navigating an environment can be successfully accomplished using either a landmark based strategy or one that relies on cardinal information (East, West, North, South). Both strategies are available to both sexes, but in a choice situation women are more likely to use landmarks, while men are more likely to rely on cardinal orientation [28]–[29]. Similar sex differences in navigational strategy are found in non-human primates and rodents [30]–[32].

Activity within ventral and dorsal streams is innately bound to cognitive development through their inherent capacity to build an associational library linking form and function in the brain [33]–[35]. For this reason, early biological or environmental influences that bias processing in one stream over the other can be expected to induce long-term effects on some aspects of cognition across species. An important biological role for androgens is indicated by numerous studies of sexual differentiation of the brain and behavior [2] [36]–[37]. Animal studies manipulating early androgen exposure have demonstrated that the male advantage in visuospatial skills is the result of a phenotypic influence of early androgen exposure rather than a direct genetic influence on visuospatial brain organization, since females treated briefly with androgens in early development showing male performance levels in adulthood [7]. Evidence to support a similar organizational role for early androgen exposure in humans is found in women with Congenital Adrenal Hyperplasia (CAH). These women are exposed to higher than normal levels of androgens in early development and subsequently perform as well as typically developing men on a number of spatial tasks that rely on the analysis of real or imagined movement, including mental rotations, the Rod and Frame Task, and targeting tasks such as throwing darts or catching a ball [38]–[39].

The content of free drawings of preschool age boys and girls offers additional indirect support for an organizing role of androgens in developing a functional bias toward processing movement [40]. Compared to typically developing girls, the drawings of girls with CAH and typically developing boys are significantly more likely to a) portray moving or mechanical objects, b) depict a three dimensional arrangement, c) use fewer and darker colors, and d) show attempts at portraying objects dynamically with their function. In contrast, the drawings of typically developing girls are more likely to a) show people, b) use more and warmer colors, and c) have objects arranged in a row without regard to realistic/relative size. These findings have a parallel in the results from studies of children's toy preferences. Typically developing boys and girls with CAH prefer toys that move, such as trucks, whereas typically developing girls prefer clothes, household items, and dolls [39]. At first glance these results appear to reflect a strong cultural bias, but this interpretation is tempered by a report of similar sex differences in the preferences for human toys in non-human primates [41].

For many of the tasks that show cognitive sex differences in humans, two performance strategies are available; one that favors a ‘bottom-up’ analysis versus one that relies on top-down analysis. In the bottom-up approach, perceptions emerge from data acquired through sensory input, in contrast to a ‘top down’ approach where perception relies on prior knowledge used to interpret that data. Bottom-up processing in higher cognitive tasks relies on both dorsal and ventral stream analysis, which is subsequently elaborated by top-down frontal analysis [42]. It is the degree to which top-down analysis is involved in the process that determines the bottom-up versus top-down distinction.

During mental rotation tasks, greater activation of bottom up processing within the dorsal stream is consistently observed in males compared to females, while greater activation of frontal circuitry is observed in women [43]–[46]. Interestingly, when activation patterns are compared in men and women with equal performance on mental rotation tasks, the sex difference in bottom-up versus top-down activation still remains [47]–[48]. The pattern suggests that women are more likely to use an analytical, top-down approach that compares and contrasts object features to solve the mental rotation problem, while males are more likely to rely on dorsal stream processing associated with mentally rotating the figures for comparison. A similar pattern of results has been observed for auditory spatial tasks, wherein males show greater bottom-up processing for sound location than females [49].

Based on this overall pattern of sex cognitive sex differences suggesting a bottom-up processing bias for movement in men and a top-down process bias in women, we reasoned that the large gender differences observed in targeting tasks might be reduced or eliminated by changing task requirements to go against the hypothesized bottom up strategy of males. To test this, we developed a computer task that employed a vertically moving ball that could be adapted for targeting or object recognition. Traditionally, ‘targeting’ is a term used to characterize tasks that require estimating the path (or vector) that an object is travelling, or will travel, as reflected in tasks such as throwing darts and catching a baseball. Most targeting studies in the literature that show sex differences involve tasks that include a significant degree of motor coordination as part of the response (e.g., throwing a ball or catching an object), although it is assumed that sex differences in task performance are primarily perceptual [12], [14]. We sought to confirm this by designing the computer task to eliminate an essential reliance on hand-eye motor coordination in the response. We also chose to use a targeting type of task for these experiments because these kinds of tasks show the largest sex differences among cognitive skills, with reported size effects ranging between 1.0 and 2.0 [14]. In addition, targeting skills have a relatively small cognitive load compared to more complex spatial tasks such as mental rotation or embedded figures, but are proposed to play an indirect role in higher-level spatial tasks that rely on abstract movement [12].

**Figure 1:**
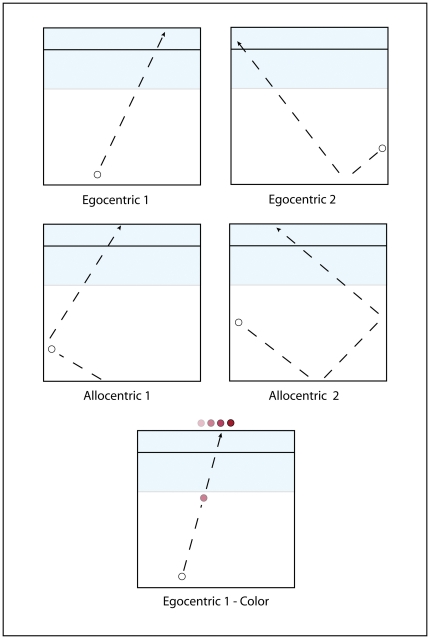
The four EVITA ball movement perspectives are shown in the top two rows. In the color shade recognition condition (represented in the bottom row), on 50% of the trials a white ball changed randomly to a shade of red, blue or yellow just before it goes under the masking shade. Participants choose the color shade from four choices on the top of the screen.

We employed three testing conditions in these experiments. The first was a targeting condition, where participants were instructed to estimate where a moving ball would intersect a horizontal line. The ball always disappeared behind a mask before reaching the intersect line. The second condition employed the same stimuli, but examined sex differences in conscious object recognition. Here, a white ball moving toward the horizontal line changed color for 100 milliseconds just before it went beneath the mask. Participants were required to identify the shade of the color to which it changed by choosing among four choices that appeared after the ball crossed the line. No targeting response was required and participants tested in this condition were given no experience with the targeting condition. These two conditions are depicted in Figure 1.

The third condition was a dual attention condition designed to require participants to prepare for both targeting and color recognition at the start of a trial. In this task, the ball randomly changed color on half of the trials, requiring participants to identify the color shade. On the trials where the ball did not change color, participants estimated the point of intersection. In this dual attention condition, we expected males to experience a degree of interference at the start of each trial because they are holding opposing strategies that depend upon whether it will be a targeting or color recognition tasks. The interference would derive from their bias toward bottom up-processing for targeting, which goes against the required top-down strategy for object recognition. Therefore, we expected male targeting performance to be relatively impaired in this condition compared to performance observed when only targeting was required. In contrast, we reasoned that a top-down strategy for targeting in females should present little interference and their performance should be similar to targeting alone. Support for these hypotheses is provided in the results of the studies described below.

## Results

### Targeting and Reaction Time (Experiment 1)

**Figure 2:**
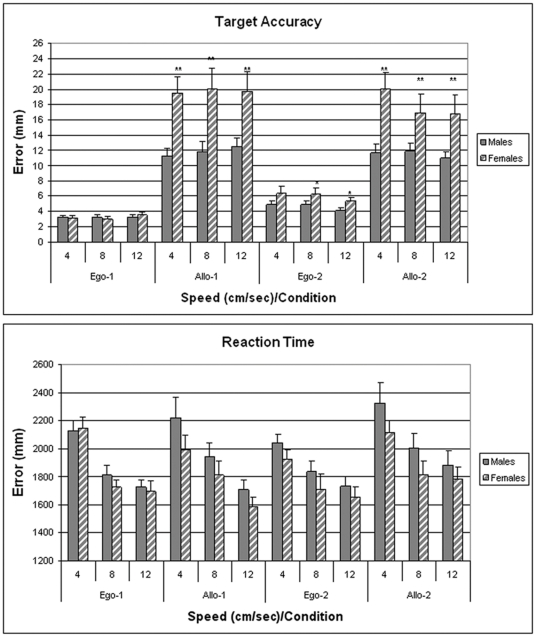
Data shown in the upper panel are the mean (±SEM) for target accuracy measured under egocentric and allocentric perspectives for 18 males and 17 females tested in Experiment 1. Data are expressed as millimeters of deviation from zero. *p<0.05, **p<0.01 from males in same condition/speed. Mean reaction times (±SEM) for the targeting response are shown the lower panel.

Preliminary analyses of the accuracy results of Experiment 1 revealed no left/right error bias, so this factor was included in subsequent analyses. Accuracy across the four conditions was analyzed using a 2 (Gender)×3 (Ball Speed)×4 (Ball Perspective) ANOVA with repeated measures over Speed and Ball Orientation. Main effects were observed for Gender (F[1,33] = 9.17; p<0.01), and Ball Orientation (F[3,99] = 88.02; p<0.0001). There was also a significant Gender X Ball Orientation interaction (F[3,99] = 7.81; p<0.001). As shown in Fig. 2, males were significantly more accurate than females in both allocentric conditions, as well as Ego-2. Effect sizes for accuracy in the Allo-1 and Allo-2 conditions ranged from 0.91 to 1.1 (Cohen's *d*).

Reaction time showed little relationship to ball perspective, but a strong inverse relationship to ball speed was observed. Analyses revealed a main effect of Speed (F[2.66] = 57.39; p<0.0001) and a Speed X Ball Perspective interaction (F[6,198] = 2.26; p<0.05). Figure 2 shows that reaction time in both sexes decreased as ball speed increased, with a steeper change across speeds in the more difficult allocentric perspectives. Covariate analysis of error and reaction times revealed no significant relationship between the two variables across ball speed or perspective.

### Color Shade Detection and Reaction time (Experiment 2)

**Figure 3:**
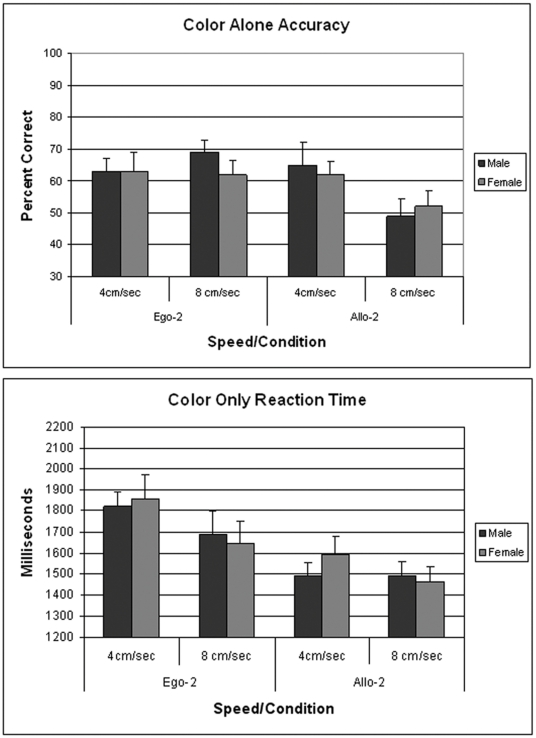
Data shown in the upper panel are the percentage of correct responses (mean ±SEM) for color discrimination of 15 females and 13 males tested in Experiment 2. Lower panel shows the mean (±SEM) reaction time for the same participants. No targeting responses were required in this condition and participants had no experience with the targeting aspects of the EVITA task.

Accuracy in discriminating the color shade was analyzed using a 2 (Gender)×2 (Ball Speed)×2 (Ball Orientation) ANOVA with repeated measures over Speed and Ball Orientation. A similar analysis was employed to analyze reaction time. The analysis of color discrimination accuracy revealed significant main effects for Ball Orientation (Ego-2 vs Allo-1: F[1,26] = 4.44; p<0.05) and ball speed (F[1,26] = 5.12; p<0.05), as well as a significant interaction between condition and speed (F[1,26] = 7.67; p<0.02). No sex effects were observed. As shown in the upper panel of Figure 3, accuracy in both sexes was similar in both conditions at the slow speed, whereas there was a significant drop in accuracy in the Allo-1 condition at the fast speed.

The analysis of reaction time revealed significant main effects for Ball Orientation (F[1,26] = 7.38; p<0.02) and Ball Speed (F[1,26] = 24.75; p<0.0001). The bottom panel of Figure 3 shows that reaction time to make a choice was faster when the balls travelled at 8 cm/sec compared to 4 cm/sec. Reaction times at both speeds were slower for the Ego-2 condition compared to the Allo-1 condition.

### Dual Attention

#### Results

**Figure 4:**
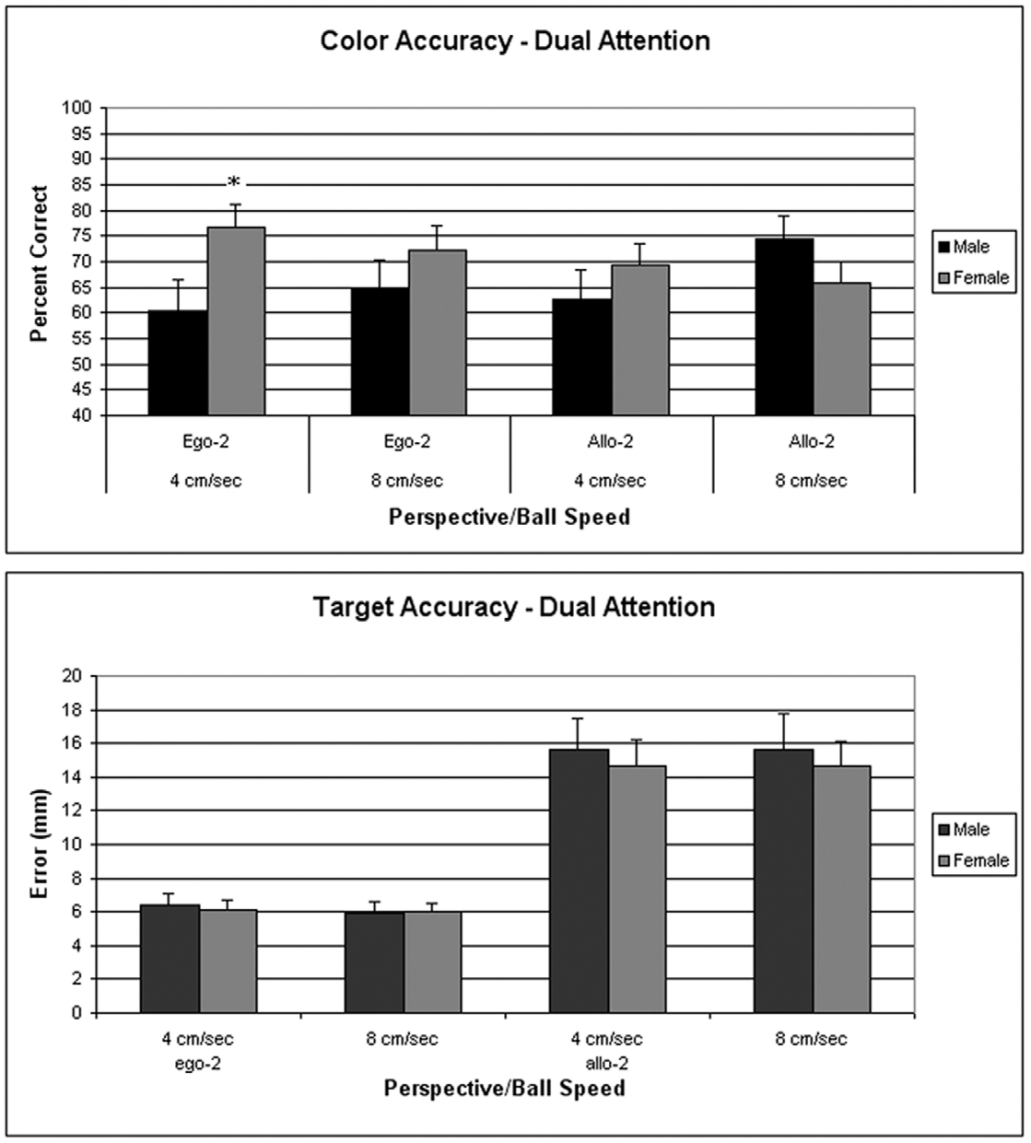
Data shown in the upper panel are the percentage of correct responses (mean ±SEM) for color discrimination in 19 males and 27 females under dual attention conditions in Experiment 3. *p<0.05 from males in same condition. Lower panel shows the mean target error (±SEM) for the same participants.

Target accuracy and reaction time were each analyzed using a 2 (Gender)×2 (Ball Speed)×2 (Ball Perspective) ANOVA, with repeated measures over Ball Speed and Ball Perspective. The analysis of target accuracy revealed only a main effect of Ball Perspective (F[1,44] = 88.35; p<0.0001). As shown in Figure 4, error rates were significantly higher at both speeds in the Allo-2 condition, compared to the Ego-2 condition. Males and females did not show any significant differences in targeting accuracy in this task. The analysis of color accuracy revealed a significant interaction between Ball Perspective and Gender (F[1,44] = 4.85; p<0.05). Figure 4 shows that female color accuracy was significantly better than male accuracy in the Ego-2 perspective at 4 cm/sec (p<0.05).

**Figure 5:**
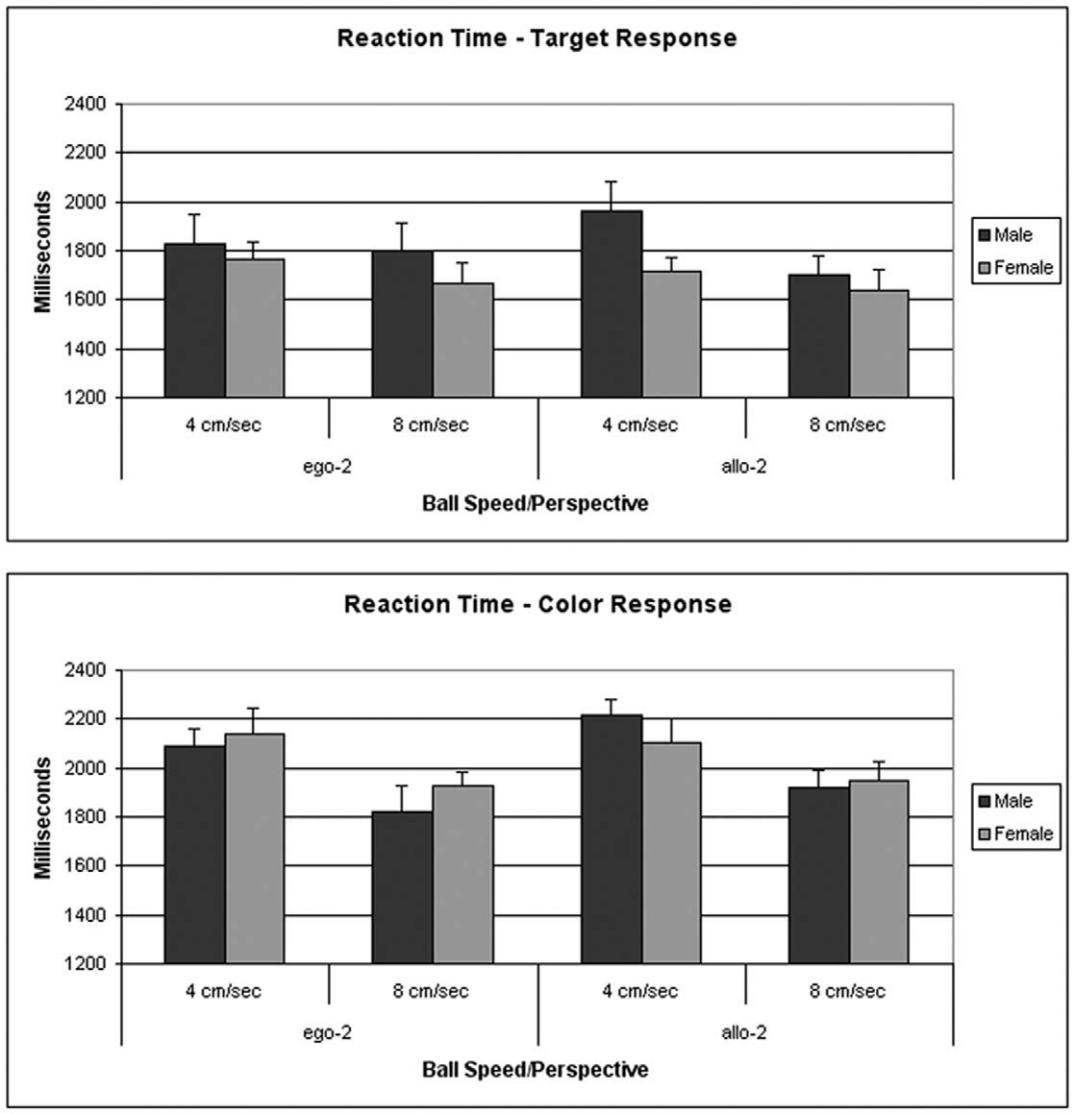
Data shown in the upper panel are the mean reaction times (±SEM) when the dual attention task required a targeting response. Data in the lower panels are the mean reaction times (±SEM) when the task required a color recognition choice. Data shown are for the 19 males and 27 females tested in Experiment 3.

Main effects for Ball Speed were observed in the analysis of the target response reaction time, (F[1,44] = 7.37; p<0.01) and the analysis of the response time to make a color choice (F[1,44] = 24.27; p<0.0001). Figure 5 shows that the reaction time in males and females decreased when ball speed was increased from 4 cm/sec to 8 cm/sec. No significant sex differences were observed in reaction time.

### Dual Attention: Within Subject Comparison across Conditions

**Figure 6:**
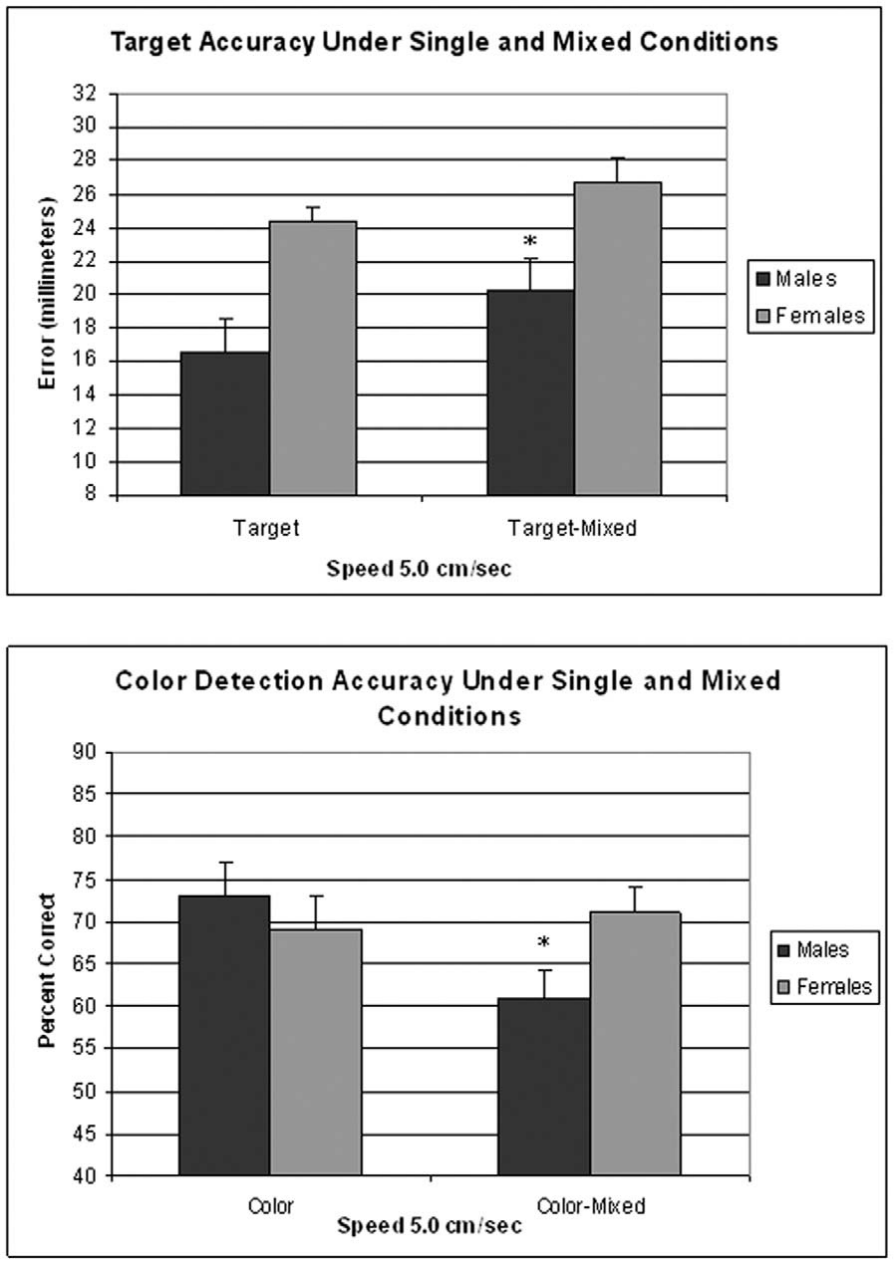
Data shown in the upper panel are the mean target error (±SEM) for targeting tested alone and under dual attention conditions. Data in the lower panels are the percentage of correct responses (±SEM) for color recognition tested alone and under dual attention conditions. Data shown are for the 15 males and 17 females tested first for color and targeting alone, followed by the dual attention condition (Experiment 4). *p<0.05 from males in the target or color alone condition.

Data were analyzed using a 2 (Gender)×2 (Color and Target Accuracy in the single condition)×2 (Color and Target Accuracy in the dual condition) ANOVA with repeated measures over the last two factors. The analysis yielded main effects for Gender (F[1,31] = 15.61; p<0.0004, Single Condition (F[1,31] = 6.36; p<0.02, and Dual Condition (F[1,31] = 199.8; p<0.0001), as well as interactions between Gender and Dual Condition (F[1,31] = 12.16; p<0.01), and Single Condition and Dual Condition (F[1,31] = 8.18; p<0.01). Overall, the analysis revealed that target accuracy was better in males under both conditions compared to females. However, males and females showed a differential response pattern under dual attention conditions compared to single testing of color and target accuracy. These effects were examined in subsequent analyses, where target and color accuracy were separately analyzed using a 2 (Gender)×2 (Condition: Dual vs Single) with repeated measures over Condition. The analysis of target accuracy yielded main effects of Gender (F[1,30] = 12.42; p<0.01) and Condition (F[1,30] = 10.63; p>0.001). Subsequent analyses revealed that male, but not female, accuracy was significantly poorer under the Dual Attention condition (F[1,14] = 7.5; p<0.02). Male color accuracy was also found to be significantly poorer under the dual attention condition as reveled by a Gender X Condition interaction (F[1,30] = 4.77; p<0.04). These results are depicted in Figure 6.

## Discussion

These studies show a large and consistent male advantage for accurately estimating the vector of a moving ball. The sex difference is present whether the ball movement has an egocentric or allocentric orientation and is not influenced significantly by slow or fast ball speeds. In Experiment 1, the size effect of the sex difference approached 1.0 in the allocentric conditions, which is in the range reported for sex differences in targeting tasks that involve physical responses such as catching or throwing [14]. These findings support the assumption that perceptual factors unrelated to motor coordination play an essential role in the sex difference in targeting performance [12], [14].

Support for the hypothesis that males are biased for processing movement over objects was found in the results from the dual attention experiments. The large sex difference in targeting performance that we observed in Experiment 1 was absent in the dual attention condition in Experiment 3. In this experiment, participants in the dual attention condition were not tested for targeting and color alone. When participants were tested for targeting and color recognition alone in Experiment 4 before being tested in the dual attention condition, the sex difference was present in the targeting alone condition, but males performed significantly worse in the dual attention condition for both targeting and color shade recognition, whereas females showed no significant change.

While these results are consistent with a male bias toward movement that was compromised by requiring them to prepare for both movement and object recognition under dual attention conditions, the color recognition results suggest that the dual attention condition causes attentional interference in males that is not observed in females. When we tested color shade recognition alone in Experiments 2 and 4, we observed no sex difference in recognition accuracy. However, in the dual attention condition in Experiments 3 and 4, male accuracy was significantly poorer than female accuracy at the slow ball speeds (4–5 cm/sec) but not the fast speed (8 cm/sec). Thus, the dual attention condition induced poorer performance for both targeting and color recognition in males, but not females. This pattern of attentional interference in males could arise from a male preference for using a bottom-up processing strategy for the targeting task, in contrast with the top-down processing required for object recognition. This would require that the bottom-up strategy co-exist with a potential top-down strategy at the onset of each trial in the dual attention condition, which would be expected to lead to some interference. If the female preference is to employ a top-down strategy for targeting, such interference would not be present because a top down analysis is required in both sexes for color shade recognition because of the *de facto* necessity of conscious analysis.

This interference interpretation is supported by results of studies showing greater top-down cortical activation in females compared with males while performing higher order spatial tasks such as mental rotation [43]–[48]. Targeting tasks require rapid analysis of real or imagined movement, which is most efficiently accomplished by a strong reliance on a bottom-up analysis associated with dorsal stream processing [52], [53]. A female preference for top-down analysis in spatial processing can also explain why females generally show poorer performance in targeting tasks [6].

Targeting accuracy in both sexes was relatively constant across the ball speeds that we used, which ranged from slow to moderately fast. Intuitively, one might expect that females would do as well as males when the ball moved slowly because it allows them time to perform a thorough assessment of ball trajectory. Yet, the results from Experiment 1 showed a consistently large sex difference in the Allocentric conditions at all three speeds tested (4.0, 8.0 and 12.0 cm/sec), with a trend toward better accuracy at the fastest speed. The fact that females did not improve with slower ball speeds is consistent with the hypothesis that females are employing a top-down strategy and may even be processing static information in the target testing environment in addition to the dynamic movement demands of the task.

A second finding that emerged from these studies is that ball speed influences the reaction time associated with the response regardless of whether the task involved a choice about movement or color recognition. Faster ball speeds led to faster reaction time in determining the intersection point of the moving ball or choosing the color shade. This relationship was observed whether the participant made a choice immediately after the event occurred or was forced to wait for an additional 2 seconds before responding. At first glance, the correlation between ball speed and reaction time appears to be consistent with the concept of embodied cognition, where simulation of ball movement is involved in the decision making process as part of the mirror neuron system (MNS) [54]–[55]. However, the reaction time results did not correlate with target or color accuracy. In addition, there was no significant sex difference in reaction time in any of the conditions studied, in spite of studies showing strong sex differences in MNS activation during the perception of movement [56]–[58]. Thus, while it appears that ball movement is an embodied cognition in both the targeting and color recognition tasks, it constitutes a channel of information that does not influence perceptual accuracy in either task.

One of the limitations of this study regarding the hypothesized sex-related processing bias is that the color recognition task was embedded within ball movement. To better address this issue, further studies are needed that employ complimentary object and movement analysis tasks, but eliminate movement within the object analysis task. Future studies are also required to define the degree to which the sex differences in the EVITA task relate to performance on higher level cognitive tasks such as mental rotations and verbal fluency.

The question of video game experience as a contributor to the present findings can also be raised since the EVITA task employs elemental skills that are inherent to video games requiring estimation of object movement vectors. Studies show that males spend more time than females playing these types of games, and that training in video games can improve female performance on a mental rotation tasks [59]–[60]. We did not control for video game experience in the present experiments, something that should be assessed in future experiments. However, we expect an effect of video game experience on EVITA task performance to be relatively small for several reasons. First, numerous targeting studies showing large sex differences favoring males have been reported between 1933 and 1986 [6], which predate the widespread use of video games. Second, the regular speed and predictable ball movement within the EVITA environment is quite different from the complexity and speed of the video game experience. Finally, we found that training 5 males and 5 females in the EVITA targeting task 4 times over a two-week period improved performance in both sexes by less than five percent (unpublished studies).

The origin of these sex differences in targeting and color recognition is unknown, as well as how they relate to sex differences in higher level tasks. While there is an extensive literature in animals and humans pointing to a phenotypic influence of androgens on the development of cognitive abilities related to spatial and movement processing, it sheds little light on how steroid actions influence the processing of objects. Because ventral and dorsal stream processing are innately tied to perceptual processing, as well as the fact that cortical androgen receptors are present during development, it might be speculated that a biological influence on the development of one reciprocally influences the development of the other. However, the picture is undoubtedly more complex since social and cultural influences related to sex role expectations also contribute to the expression of human cognitive sex differences [61].

## Methods

### Participants

Right-handed undergraduate students from San Diego State University were granted class credit or given a $5 Starbucks card for their participation. A demographic questionnaire that included information about current medications, handedness, and medical history was used to exclude participants with history of head injury, attention deficit disorder, medications that could impair attention, or neurological conditions. The age of participants ranged from 18–26. Total number of participants was 141 (74 females, 67 males). All procedures were reviewed and approved by The Committee on Protection of Human Subjects at San Diego State University.

### Task Overview: Evaluation of Variability in Targeting Aptitude (EVITA)

The computer task employs a ball moving toward a horizontal intersect line within a common visual environment. Either targeting or color recognition can be measured on a given trial. In all conditions, there is a masking screen behind which the ball disappears before it crosses the line. The program is a Flash-based application that runs on a personal computer, with a task area that is 22.5 cm wide×15 cm high. The intersect line is 17 cm from the bottom and a blue masking screen is set at 4 cm or 5 cm below the intercept line, depending upon the experiment described in the methods below. After the ball disappears beneath the masking screen it does not re-appear during the trial. Ball speed is set between 2.2 and 12.0 cm/sec depending upon the experiment, but is always constant for a given set of trials within a testing condition. Participants were seated in front of the screen at a distance of 18 inches, with a visual angle of 1.25 degrees for the ball.

### Ball Movement Perspective

EVITA provided two egocentric and two allocentric testing perspectives for ball movement (shown in Figure 1) based on evidence that allocentric and egocentric perspectives involve unique neural circuits [50]–[51]. In the Egocentric conditions, the intersect vector is estimated from the bottom of the screen to the intersect line. In the allocentric conditions the intersect vector is estimated from a sidewall to the intersect line. In the Ego-1 condition, the ball starts from the bottom of the screen and moves toward the intersect line, randomly varying across a 45 degree range. In the Ego-2 condition, the ball starts from a sidewall and moves to the bottom of the screen, where it bounces up toward the intersect line. In the Allo-1 condition, the ball starts from the bottom of the screen and bounces off a sidewall toward the intersect line. In the Allo-2 condition, the ball starts from a side wall, bounces off the bottom of the screen to the other side wall, where it bounces toward the intersect line. In the Ego-1 and Allo-1 conditions, the ball trajectory was programmed to randomly start toward the left or right side of the screen. In the Ego-2 and Allo-2 conditions, the starting point was randomly set to occur approximately 50% of the time from the left or right side wall.

### Targeting Task

When target estimation is required, a paddle appears on the horizontal line after the ball has crossed through it and the participant moves the paddle to the estimated point of intersection and clicks. The program measures error in millimeters to the right or left of where the center of the ball intersected the line. Reaction time is measured from the time that the paddle appears to make the estimation. Depending upon the experimental condition, the paddle appears immediately after the center of the ball intersects the line, or after a delay of two seconds.

### Object Recognition Task

When the task requires identifying the color shade, the moving ball changes from white to a shade of yellow, blue, or red for 100 milliseconds immediately prior to disappearing beneath the masking screen. After the ball has passed through the intersect line, four color boxes appear at the top of the EVITA screen, each showing a different shade of the color to which the ball changed. The participant clicks on the box showing the shade they saw. An example is shown in the bottom row of Figure 1. No paddle appears on the intersect line.

### Dual Attention Task

The task is designed to create a dual attention condition that requires participants at the start of each trial to prepare for both color discrimination and targeting accuracy, depending upon whether the ball changes color. On 50% of the trials within a condition, the program randomly changes the white ball to one of 4 shades of red, blue or yellow before the ball moves behind the masking screen. If the ball changes color, the color boxes appear at the top of the frame. If the ball remains white, the paddle appears on the intersection line and the participant estimates the intersection point.

### Data Analyses

Ten trials were presented to participants in the targeting or color conditions when they were tested alone. In the dual attention task, twenty trials were presented, with color and targeting conditions randomly occurring ten times. Pilot studies revealed that errors in the targeting condition were significantly greater on the first trial compared to the mean. However, over the ten trials, occasional outliers in targeting accuracy or reaction time also occurred in some participants, likely due to attention lapses. Therefore, we adopted a standard procedure of rank ordering the accuracy scores (distance error), with their associated reaction times, and used the mean of the top 80% in accuracy for analysis. Thus, ranking was only for accuracy scores, with reaction times for a given score always retained.

For color accuracy, which consisted of a right/wrong score on each trial, the mean of correct choices and reaction time for all trials was used for analyses, with the exception that reaction times greater than two standard deviations from the mean were eliminated. This resulted in the elimination of less than 0.5% of the total responses. Data were analyzed using BMDP statistical programs for correlations and ANOVA with repeated measures. Planned comparisons were conducted using a Bonferroni correction.

### Design of Experiments

Since EVITA is a novel task, we first tested targeting and color alone to control for potential interactions when we tested participants for the dual attention task. The targeting alone experiment was also designed to establish whether or not it would produce a sex difference in line with other types of targeting tasks that involve motor coordination [14].

The four experiments listed below tested the performance of separate groups of naïve males and females in the targeting task (Exp. 1) and the color recognition task (Exp. 2). The hypothesis of a male bias toward movement was tested in the dual attention task using a between-subject design that included two ball speeds (Exp. 3). The final experiment employed a within-subject design that compared the performance of males and females across all three conditions.

### Experiment 1. Effect of Egocentric/Allocentric Perspective and Ball Speed on Targeting Accuracy and Reaction Time

Targeting accuracy and reaction time were tested using three ball speeds of 4.0, 8.0 and 12.0 cm/sec. Each participant was presented with 10 trials at each speed in each of the four perspective conditions. The testing order was Ego-1, Allo1, Ego-2, Allo-2, with speed increasing after each set. The masking area was set at 4 cm below the intersect line. There was a paddle delay of 2000 milliseconds from the time the middle of the ball crossed the intersect line and participants had 3000 milliseconds to respond after the appearance of the paddle.

Nineteen males and nineteen females were tested. Each was given 5 practice trials at the start using the Ego-1 condition with no masking area and a 4 cm/sec ball speed. This provided the opportunity to see the ball cross the intersect line, as well as practice in moving the paddle to the intersect point.

### Experiment 2. Color Shade Discrimination

The ability to discriminate a brief color change in a white ball was measured in 15 females and 13 males. Participants were given 5 practice trials at the 4 cm/sec ball speed using the Ego-1 condition to familiarize them with the testing conditions. The Ego-2 and Allo-1 conditions were for testing. The order of presentation for all subjects was Ego-2 (4 cm/sec), Allo-1 (4 cm/sec), Ego-2 (8 cm/sec), Allo-1 (8 cm/sec). Ten trials were presented for each condition at each speed. The white ball always changed color for 100 milliseconds, just prior to passing beneath the masking screen, which was 4 cm below the intersect line. The color boxes appeared 2000 milliseconds after the ball passed through the intersect line. Color accuracy and reaction time were measured on each trial.

### Experiment 3. Effect of Dual attention and Ball Speed on Target and Color Accuracy

We tested 19 males and 27 females in the color/target dual attention task that primed participants for both object and movement processing at the start of each trial. Participants were given 5 practice trials at the 4 cm/sec ball speed using the Ego-1 condition to familiarize them with the testing conditions. For the experiment that followed, 20 random trials were presented under each ball orientation and speed, with the set split equally between color and targeting. The masking screen was 4 cm below the intersect line, and the paddle or the color boxes appeared 2000 milliseconds after the ball passed through the intersect line. The order of presentation for all subjects was Ego-2 4 cm/sec, Allo-2, 4 cm/sec, Ego-2 8 cm/sec, and Allo-2 8 cm/sec. Reaction time was measured for both target and color responses.

### Experiment 4. Within-Subject Comparison of Target and Color Accuracy Tested Alone and Under Dual Attention Conditions

This experiment examined whether the results of Experiment 3 were related to a lack of experience with the targeting and color recognition tasks. We conducted a within-subject comparison where 17 females and 15 males were tested first in the color alone and the target alone conditions, and finally in the color/target dual attention task. The order of presentation for the color alone and target alone was counterbalanced within sex, and the dual attention was always the last task presented. We used the Allo-1 perspective at a ball speed of 5.0 cm/sec. Ten trials each were presented for the color alone and target alone conditions. For the dual attention task, 20 trials were presented, which were split randomly between color shade recognition and targeting. The masking screen was 4 cm below the intersect line, and the paddle or the color boxes appeared 2000 milliseconds after the ball passed through the intersect line. All participants were given 5 practice trials on the color alone and the target alone conditions at the 3.5 cm/sec ball speed using the Ego-1 condition immediately before testing.
